# Perinatal and psychosocial circumstances associated with risk of attempted suicide, non-suicidal self-injury and psychiatric service use. A longitudinal study of young people

**DOI:** 10.1186/1471-2458-11-875

**Published:** 2011-11-18

**Authors:** Robert Young, Vincent Riordan, Cameron Stark

**Affiliations:** 1 MRC Social and Public Health Sciences Unit, University of Glasgow, 4 Lilybank Gardens, Glasgow G12 8RZ, Scotland, UK; 2 West Cork Mental Health Services, HSE. Bantry, Co. Cork, Ireland; Centre for Rural Realth Research and Policy, Inverness, Scotland, UK; 3 NHS Highland, Inverness, Scotland, UK, Centre for Rural Health Research and Policy, Inverness, Scotland, UK

## Abstract

**Background:**

Past studies using large population based datasets link certain perinatal circumstances (birth weight, parity, etc) with mental health outcomes such as suicide, self-harm and psychiatric problems. Problematically, population datasets omit a number of social confounds. The aim of this study is to replicate past research linking perinatal circumstances and mental health (suicidality and use of psychiatric services) and to determine if such associations remain after adjusting for social circumstances.

**Methods:**

A longitudinal school-based survey of 2157 young people (surveyed at age 11, 13, 15) followed up in early adulthood (age 19). At age 11 parents of participants provided information about perinatal circumstances (birth weight, birth complications, etc.) and psychiatric service use. Participants provided data about their mental health at age 15 (attempted suicide, suicidal thoughts) and at ages 19 (self-harm, psychiatric service use). In addition, data were collected about their social and psychosocial circumstances (gender, deprivation, religion, sexual behaviour, etc.).

**Results:**

Predictably, social factors were linked to mental health outcomes. For example, those with same sex partners were more likely (OR 4.84) to self-harm than those without a same sex partner. With a single exception, in both unadjusted and adjusted models, perinatal circumstances were not or only marginally associated with mental health outcomes. The exception was the number of birth complications; young people with two or more complications were approximately 2-3 times more likely than those without complications to use psychiatric services.

**Conclusions:**

While we failed to replicate results found using large population based datasets, some of our results are compatible with prior research findings. Further, evidence from this study supports the influence of perinatal circumstances (birth complications) on later psychiatric problems, or at least higher than expected contact with psychiatric service.

## Background

Mental health in adulthood may be significantly influenced by earlier life environments [1,2]. The importance of the childhood and adolescent environment has long been recognised. Attachment theory [3] emphasises the importance of the earliest relationships, and robust associations have been demonstrated between mental ill-health in adulthood and such childhood adversities as parental separation [4] and childhood maltreatment or abuse [5-9]. In more recent years variables from the perinatal environment have also been demonstrated as associates of adult mental health outcomes. Adverse outcomes have been linked with factors such as antenatal maternal malnutrition [10], antenatal maternal stress [11], infant malnutrition [12], low birth weight, younger maternal age and increasing birth order [13-16]. These observations, in particular those involving the antenatal environment, suggest a possible biological basis, possibly involving epigenetic developmental processes [17].

Most mental ill-health has a complex multifactorial aetiology and any environmental associations may be of clinical or public health importance in that they may indicate possible areas for preventative intervention. However, because of the possibility of unidentified confounding variables, it is important to attempt to distinguish between factors which, although linked to increased risk, do not in themselves mediate that risk (risk indicators), as opposed to factors which directly contribute to the causal pathway leading to the development of mental illness (risk mediators). For example, a number of studies have suggested that much of the association between parental separation and later adverse mental health outcomes can be accounted for by other psychosocial variables, such as socioeconomic deprivation, exposure to parental conflict and poor child-parent relationships [18-20].

The relative importance of very early influences, such as those occurring in the perinatal period, compared with later variables, such as adolescent or early adult circumstances is unclear [21]. Much of the existing evidence regarding perinatal variables relies on large population based datasets. Whereas these have the advantage of avoiding recall bias and yielding high statistical power, they may exaggerate the significance of variables such as maternal demographics, on which data are readily available, whilst failing to control for plausible confounders, such as breastfeeding or family structure, on which data (from large datasets) tends to be poor or absent.

### Aims

The aim of the study is to examine, using self reported data, the association between perinatal and psychosocial risk factors and mental health outcomes, thereby testing the hypothesis that both perinatal circumstances and more chronologically proximate psycho-social circumstances are risk mediators, (rather than indicators) of mental ill-health in early adulthood. The study allowed a number of variables from different stages of development to be brought together in a single multivariable analysis whilst also replicating previous studies using an alternative study design.

## Methods

The material for the study is drawn from a Scottish longitudinal community health and lifestyle survey of young people, administered first in-school via questionnaire (ages 11, 13, and 15), with an additional parental questionnaire at age 11, and then in the post-school period by nurse interview at age 19. The focus here is on mental health outcomes at age 15 (1999) and age 19 (2003) within the framework of the 'West of Scotland 11 to 16 Study/16+' [22]. The study received approval from Glasgow University's Ethics Committee, participating Education Authorities and schools, and informed consent was obtained from the parents of all participants via 'opt-out' consent forms at ages 11, 13 and 15, verbal consent from participants at each wave and written consent at age 19.

Due to the school-based nature of the sample the sampling scheme involved several elements to ensure a representative sample at both the primary and secondary school stages [23]. Briefly, the survey used a reverse sampling procedure which randomly selected 43 secondary schools stratified by religious denomination and deprivation, with a separate stratum for independent vs. local authority run schools. These 43 secondary schools were used to select a random sample of 135 primary schools, comprising 'feeder schools', together with those making a high number of placing requests. From these primary schools, classes were randomly selected with all pupils in the classes eligible to participate. Of the 2793 pupils who attended the 43 targeted secondary schools, 2586 (93%) participated in the baseline (age 11) survey. At age 13, the number of participants reduced to 2371 (85%), and by 15 to 2196 (79%). At age 15, 1,860 (67%) of respondents completed a psychiatric interview [24], which included questions about suicidal thoughts and attempts. As expected losses in the post-school period substantially reducing the sample size at age 19 to 1256 (45%). Full details of the sampling strategy are available elsewhere [23].

At age 11 the sample was representative (in terms of sex and social class composition) of 11 year olds in the study area [25]. Differential attrition made later waves less representative, with attrition greater among lower social class groups, school truants, pupils of lower ability and with greater emotional and behavioural problems. To compensate for these biases, a weighting scheme was derived [25]. Use of these weights did not substantively alter any of the results presented here. The data used in this paper refer to 2196 pupils in their final year of compulsory education in 43 mainstream secondary schools in the Glasgow area, 1256 of whom provided information when aged 19. Parents provided information on perinatal circumstances such as maternal age and birth weight and on pupils' religious background and family socioeconomic status via a questionnaire in the first wave (age 11) of the study. The final sample included 16 twin births, but excluded adopted children (n = 39), reducing the sample to 2157.

### Measures

In 1996 (pupils aged 11) parents were asked questions about the child's birth history and perinatal circumstances. This included questions on maternal parity, categorised as first or later born; family size (including parents), categorised as 2 or more family members; subjective recall of the size of the baby (small, average, or large); birth weight (in grams), categorized <2500, 2500-3249, 3250-3749, or 3750g+; number of birth complications (e.g. breech birth, pre-eclampsia, etc), categorized none, one, two or more; birth spacing (both prior to and post index birth), categorised as under 2 years, 2-5 years, or 6+ years between births (or singleton); maternal age, categorised age 15-19, 20-24, 25-29, 30-34, or 35-46. Finally, parents provided information on breastfeeding practices, categorised as none, breastfeeding for less than six months, or six months or longer. Whenever feasible we derived categories compatible with established cut-points or expert recommendations. For example, in relation to the six month cut-point for breastfeeding, the American Academy of Pediatrics recommendation is '*exclusive breastfeeding is sufficient to support optimal growth and development for approximately the first 6 months of life*' [26].

Several background factors relevant to either socio-demographic circumstances or psychiatric outcomes at age 11 and age 15 were recorded. At age 11 an area deprivation score, range 1 (least) to 7 (most deprived), was derived from pupils' postal codes using the 'Carstairs' [27] index, a standard measure based upon census data. Social class of the head of household was derived from parental questionnaires completed at wave one (age 11), coded using the standard UK classification system [28] and categorized as non-manual or manual. Religious affiliation was obtained from parents and categorized, Church of Scotland (Protestant), Catholic, Muslim, other (Jewish, Methodist, Baptist, etc) and 'none, atheist/agnostic'. At age 15, pupil's family structure was coded as 2-parent, 1-parent, reconstituted (one 'birth' parent and new partner) or other (relative, foster parent, or other carer). Principal component (varimax) analysis of the (age 15) 8-item Brief Parental Bonding Instrument [29], produced two scales representing perceived (low) parental care, e.g. 'My parents help me as much as I need' (reversed) and (high) control, e.g. 'My parents treat me like a baby'.

Sexual orientation is an established psychosocial risk factor for suicide and other psychiatric disorders and early gender nonconformity is a strong predictor of future sexual orientation [30,31]. Gender diagnosticity (GD) is an established measure of gender nonconformity, previously measured in this cohort at age 15 and linked with poor psychological wellbeing [30]. GD score is the probability of belonging to a particular gender, as predicted by the logistic regression of multiple items of gendered behaviours, interests and hobbies. The result is a score (0-1 scale) of how 'typically' male or female an individual is (within a limited domain) compared to their peers. An arbitrary cut-point of 0.1 (10%) has been used to indicate 'gender conformist' (top 10%), 'Gender typical' (middle 80%) and gender nonconformists (bottom 10%) respectively. At age 19, participants were asked 'Have you ever had any kind of sexual experience or sexual contact with someone of the same gender as yourself' and asked to response 'yes' or 'no' using a 'show card procedure' designed to reduced response bias. While not a comprehensive measure of sexual preference, this item is arguably a good indicator of same-sexual orientation and behaviour.

Several dichotomous mental health outcomes were reported at ages 15 and 19. At age 15 participants were asked if they had ever seriously thought about taking their own life and if they had attempted to do so, categorised as suicidal thoughts and attempted suicide respectively, with a positive response to either question classified as 'suicide risk'. At age 19 participants were asked if they had ever 'tried to hurt or harm yourself deliberately' and to indicate which method(s) the had used, such as cutting, scratch or scoring or burning; a positive response was classified as Nonsuicidal Self-Injury NSSI. To assess previous psychiatric problems, parents were asked about the child's use of psychiatric services *until *age 11 and at age 19 participants were asked about their use of psychiatric services *since *age 11. Due to both budget and ethical constrains suicidality was only measured at age 15 and 19', self-harm at age 19 and psychiatric contact only measured at age 11 and 19.

### Statistical analysis

The analysis used logistic regression to determine the association between perinatal and psychosocial circumstances and age 15 and age 19 psychiatric outcomes. Analysis was conducted both unadjusted and mutually adjusted for covariates. We constructed weights to compensate for differential attrition (21), but use of these weights did not alter results. The influence of missing data was further explored by comparing results for models using complete data only and multiple imputation methods. Multiple imputation was implemented using the STATA 'ice' procedure and included all variables from the relevant model, with separate imputation runs for every analysis. Birth size was included as a supplementary variable, because it contained information useful in estimating birth weight. Categorical variables were imputed using logit or multiple logit, continuous variables using regression and deprivation using ordinal logit commands. Ten imputed datasets were used to calculate the final combined estimates. Although the results for each method were not substantively different, we report results based on multiple imputation. Results from all other models are available upon request.

## Results

### Univariate results

Table 1 reports descriptive statistics and the number of cases with missing data for the perinatal covariates; table 2 reports these for psychosocial covariates and psychiatric outcomes. Given the age and nature of the cohort, the observed pattern of results is typical for this type of survey data.

**Table 1:** Descriptive statistics for perinatal covariates

Perinatal covariates ^all @11p^	N	(%)
**Maternal parity**		
0 first born	989	45.9
*1 second born	784	36.3
*2+ third or later	384	17.8
**Family size (total at age 11) +Twins**		
2	247	11.5
*3	1001	46.4
*4+	909	42.1
**Birth size [MV = 20]**		
Small	293	13.7
Average	1458	68.2
Large	386	18.1
**Birth weight (g) [MV = 96]**		
<2500	133	6.5
2500-3249	719	34.9
3250-3749	801	38.9
3750+	408	19.8
**Birth complications [MV = 15]**		
0	1499	70.0
1	360	16.8
*2	189	8.8
*3 or more	94	4.4
**Birth spacing - Prior birth**		
Under 2 years	346	16.0
2-5 years	502	23.3
6+ years or singleton	1309	60.7
**Birth spacing - Next birth**		
Under 2 years	429	19.9
2-5 years	510	23.6
6+ years or singleton	1218	56.5
**Maternal age (years) [MV = 79]**		
*15-19*	139	6.7
*20-24*	626	30.1
*25-29*	764	36.8
*30-34*	421	20.3
*35-46*	128	6.2
**Breastfeeding [MV = 12]**		
None	1333	62.1
Less than 6 months	463	21.6
6 months or more	349	16.3

Baseline N = 2157, final sample excludes adopted children i.e. only natural mother.

MV = missing values; * Adjacent category collapsed for analysis; +Twins = includes 16 twin births.

^@11p ^= parental figure provided information when participants age 11.

**Table 2:** Descriptive statistics for psychosocial covariates and psychiatric outcomes.

Social and demographic covariates	N	(%)
**Sex ^@11^**		
Female	1076	49.9
Male	1081	50.1
**Social Class ^@11p ^[MV = 376]**		
Manual	860	48.3
Non-manual	921	51.7
**Family structure age 15 ^@15 ^[MV = 288]**		
2-parent	1410	75.4
1-parent,	274	14.7
Reconstituted/Other	185	9.9
**Same sex partner by 19 ^@19 ^[MV = 1066]**		
No	1045	95.8
Yes	46	4.2
**Gender nonconforming behaviour at age 15** **(Gender Diagnosticity) ^@15 ^[MV = 289]**		
Gender conformist (10%)	179	9.6
Gender typical (80%)	1500	80.3
Gender nonconformist (10%)	189	10.1
**Religion ^@11p ^(MV = 19) **		
Protestant	913	42.7
Roman Catholic	721	33.7
*Muslim/Islam	57	2.7
*Other (Baptist, Jewish, etc)	125	5.8
None/Atheist/Agnostic	322	15.1

**Psychiatric outcomes**	**N**	**(%)**

**Suicidal thoughts past year at age 15 **^**@15 **^**[MV = 522]**		
No thoughts	1513	94.3
Thoughts	92	5.7
**Attempted suicide by age 15 ^@15 ^[MV = 522]**		
No attempt	1511	94.1
Attempt	94	5.9
**Risk: Any ideation/attempt by age 15 ^@15 ^[MV = 522]**		
None	1460	91.0
Attempt or ideation	145	9.0
**NSSI by age 19 ^@19 ^[MV = 1066]**		
No NSSI	1013	92.9
NSSI	78	7.1
**Psychiatric services by age 11 ^@11p ^[MV = 11]**		
Not used	2064	96.2
Used	82	3.8
**Psychiatric services since age 11 ^@19 ^[MV = 1066]**		
Not used	1047	96.0
Used	44	4.0
**Psychiatric services ANY **^**@11p, @19 **^**[MV = 1070]**		
Not used	1017	93.6
Used	70	6.4

**Continuous covariates**	**M**	**SD**

**Area deprivation (depcat) **^**@11 **^**[MV = 299]**	4.11	1.90
**(Lack of) Parental Care **^**@15 **^**[MV = 288]**	5.59	1.59
**Parental Control **^**@15 **^**[MV = 288]**	7.85	1.09

Baseline N = 2157, final sample excludes adopted children i.e. only natural mother.

MV = missing values; * Adjacent category collapsed for analysis.

^@11 ^= participant provided information at age 11; ^@15 ^= information provided at age 15; ^@19 ^= information provided at age 19; ^@11p ^= parental figure provided information when participants age 11.

### Suicidality and self-harm

Table 3 shows the unadjusted associations between covariates and rate of attempted suicide, suicide risk (either ideation or attempt) at age 15 and NSSI by age 19. As expected, several psychosocial covariates are associated with significant increases in the odds in attempted suicide, suicide risk or NSSI. For example, compared to males, females show approximately a threefold increase in the odds of attempting suicide or having suicidal thoughts, although adjusting for covariates slightly attenuates this. Being part of a reconstituted family was also a risk factor for all suicidality and NSSI outcomes and lack of parental care was another. Compared to the 'gender typical' group, 'gender nonconformists' had a twofold increase in the odds of attempted suicide or NSSI, although this was non-significant in the adjusted model. In the unadjusted model, young people who had at least one same sex partner show nearly a fivefold increase in odds of NSSI, compared to those who never had a same sex partner; this association remained strong in the adjusted model (OR = 3.85). Perinatal variables were unrelated or only marginally associated with suicidality or self-harm.

**Table 3:** Associations (odds ratios) between social, demographic, perinatal factors and suicide risk or self-harm.

	Suicide attempt by age 15		Suicide risk: Suicidal ideation/attempt by age 15		NSSI by 19	
			
Covariates	Unadjust	Adjusted	Unadjust	Adjusted	Unadjust	Adjusted
**Psychosocial **						

**Sex ^@11^**						
Male	1.00	1.00	1.00	1.00	1.00	1.00
Female	**3.20 (1.93-5.30)**	**3.06 (1.79-5.22)**	**2.77 (1.91-4.03)**	**2.69 (1.83-3.96)**	1.32 (0.89-1.97)	1.25 (0.82-1.89)
**Social class ^@11p^**						
Non-manual	1.00	1.00	1.00	1.00	1.00	1.00
Manual	1.39 (0.86-2.25)	1.20 (0.71-2.03)	1.20 (0.85-1.69**)**	1.03 (0.66-1.59)	0.90 (0.55-1.45)	0.85 (0.49-1.49)
**Family structure ^@15^**						
2-parent	1.00	1.00	1.00	1.00		1.00
1-parent,	1.48 (0.80-2.73)	1.25 (0.64-2.44)	1.34 (0.80-2.26**)**	1.20 (0.67-2.15)	1.43 (0.77-2.65)	1.34 (0.70-2.56)
reconstituted/Other	**3.21 (1.93-5.34)**	**2.37 (1.30-4.34)**	**2.51 (1.61-3.92)**	**2.10 (1.27-3.48)**	**2.84 (1.43-5.65**)	**2.47 (1.20-5.10)**
**Same sex part ^@19^**						
No	1.00	1.00	1.00	1.00	1.00	1.00
Yes	2.19 (0.68-7.07)	1.39 (0.33-5.78)	1.48 (0.59-3.72**)**	1.03 (0.33-3.26)	**4.84 (2.24-10.46**)	**3.85 (1.67-8.87)**
**Gender conform ^@15^**						
Gender typical	1.00	1.00	1.00	1.00	1.00	1.00
Gender conformist	0.79 (0.34-1.81)	0.82 (0.36-1.89)	0.82 (0.42-1.62**)**	0.87 (0.44-1.72)	0.95 (0.51-1.78)	1.01 (0.51-1.99)
Gender nonconformist	**1.89 (1.06-3.35)**	1.65 (0.80-3.41)	1.24 (0.72-2.16**)**	1.12 (0.58-2.19)	1.84 (0.91-3.71)	1.38 (0.59-3.22)
**Religion ^@11p^**						
Protestant	1.00	1.00	1.00	1.00	1.00	1.00
Roman Catholic	0.92 (0.55-1.52)	0.89 (0.52-1.54)	0.88 (0.58-1.32**)**	0.88 (0.57-1.38)	0.77 (0.48-1.22)	0.81 (0.50-1.34)
Other (Baptist, etc)	0.99 (0.49-1.99)	1.15 (0.54-2.44)	0.66 (0.34-1.28**)**	0.72 (0.36-1.47)	1.17 (0.64-2.11)	1.28 (0.63-2.60)
None/Atheist/Agnostic	1.09 (0.57-2.08)	0.97 (0.49-1.91)	0.91 (0.54-1.56**)**	0.84 (0.48-1.46)	1.11 (0.54-2.29)	1.01 (0.46-2.21)

**Area deprivation ^@11^**	1.09 (0.96-1.23)	1.04 (0.89-1.20)	1.04 (0.95-1.14**)**	0.99 (0.88-1.12)	1.01 (0.90-1.13)	1.02 (0.88-1.17)
**Lack of par Care ^@15^**	**1.33 (1.19-1.49)**	**1.30 (1.15-1.47)**	**1.34 (1.21-1.49)**	**1.33 (1.18-1.49)**	**1.32 (1.18-1.48**)	**1.30 (1.16-1.46)**
**Parental control ^@15^**	1.15 (0.96-1.37)	1.18 (0.98-1.43)	1.10 (0.94-1.29**)**	1.13 (0.96-1.33)	0.99 (0.82-1.19)	0.97 (0.79-1.18)

**Perinatal ^all @11p^**						

**Maternal parity**						
**0**	1.00	1.00	1.00	1.00	1.00	1.00
1 or more	1.11 (0.70-1.76)	1.67 (0.60-4.65)	1.06 (0.70-1.60**)**	1.44 (0.65-3.20)	0.77 (0.45-1.32)	0.74 (0.28-1.92)
**Family size **						
2	1.00	1.00	1.00	1.00	1.00	1.00
3 or more	0.78 (0.45-1.34)	1.03 (0.47-2.29)	0.96 (0.58-1.58**)**	1.19 (0.61-2.30)	1.08 (0.35-3.34)	1.50 (0.39-5.85)
**Birth weight (g)**						
<2500	1.44 (0.63-3.30)	1.46 (0.56-3.84)	1.30 (0.65-2.58**)**	1.39 (0.66-2.94)	0.70 (0.33-1.50)	0.67 (0.28-1.64)
2500-3249	1.12 (0.71-1.77)	1.13 (0.70-1.84)	1.08 (0.75-1.55**)**	1.09 (0.74-1.61)	**0.57 (0.33-1.00**)	0.57 (0.30-1.10)
3250-3749	1.00	1.00	1.00	1.00	1.00	1.00
3750+	0.74 (0.42-1.30)	1.04 (0.58-1.88)	0.81 (0.51-1.29**)**	1.03 (0.63-1.70)	0.71 (0.40-1.23)	0.80 (0.44-1.48)
**N birth complications**						
0	1.00	1.00	1.00	1.00	1.00	1.00
1	0.88 (0.49-1.58)	0.97 (0.53-1.78)	0.71 (0.43-1.16**)**	0.74 (0.44-1.25)	1.10 (0.62-1.96)	1.21 (0.65-2.27)
2 or more	0.90 (0.47-1.74)	1.00 (0.49-2.02)	0.84 (0.50-1.40**)**	0.90 (0.51-1.58)	1.36 (0.74-2.51)	1.72 (0.91-3.28)
**Birth spacing (prior)**						
Under 2 years	1.00	1.00	1.00	1.00	1.00	1.00
2-5 years	0.82 (0.45-1.49)	0.88 (0.46-1.68)	1.00 (0.59-1.67**)**	1.06 (0.61-1.84)	0.86 (0.50-1.50)	0.86 (0.46-1.59)
6+ years or singleton	1.02 (0.60-1.72)	0.91 (0.48-1.74)	1.03 (0.64-1.65**)**	1.00 (0.58-1.74)	0.68 (0.43-1.09)	0.69 (0.39-1.22)
**Birth spacing (later)**						
Under 2 years	1.00	1.00	1.00	1.00	1.00	1.00
2-5 years	0.73 (0.39-1.38)	0.81 (0.42-1.57)	0.80 (0.46-1.40**)**	0.85 (0.48-1.52)	0.66 (0.31-1.42)	0.73 (0.32-1.69)
6+ years or singleton	0.92 (0.55-1.55)	1.25 (0.54-2.93)	0.95 (0.58-1.57**)**	1.15 (0.54-2.46)	0.96 (0.59-1.56)	0.67 (0.26-1.70)
**Maternal age (years)**						
*15-19*	1.84 (0.92-3.67)	1.25 (0.59-2.66)	1.68 (0.90-3.11**)**	1.27 (0.65-2.49)	0.92 (0.31-2.74)	0.86 (0.28-2.65)
*20-24*	1.25 (0.79-2.00)	1.00 (0.58-1.71)	1.20 (0.81-1.78**)**	1.05 (0.66-1.66)	1.11 (0.65-1.89)	1.04 (0.58-1.88)
*25-29 *	1.00	1.00	1.00	1.00	1.00	1.00
*30-34*	0.82 (0.45-1.51)	0.86 (0.45-1.66)	1.04 (0.66-1.64**)**	1.04 (0.63-1.73)	1.19 (0.73-1.96)	1.23 (0.74-2.07)
*35-46*	1.09 (0.47-2.53)	1.29 (0.51-3.24)	1.25 (0.62-2.53**)**	1.44 (0.68-3.08)	1.03 (0.45-2.37)	1.02 (0.38-2.74)
**Breastfeeding**						
None	1.00	1.00	1.00	1.00	1.00	1.00
Less than 6 months	0.78 (0.43-1.43)	0.94 (0.49-1.79)	0.67 (0.40-1.13**)**	0.75 (0.43-1.33)	0.90 (0.51-1.60)	0.96 (0.48-1.88)
6 months or more	0.63 (0.34-1.16)	0.87 (0.42-1.80)	0.75 (0.47-1.18**)**	0.87 (0.52-1.45)	0.89 (0.48-1.68)	0.90 (0.47-1.74)

Significant odds ratios (p = 0.05 or lower) are shown in bold. Final sample with multiple imputation = 2157.

^@11 ^= participant provided information at age 11; ^@15 ^= information provided at age 15; ^@19 ^= information provided at age 19; ^@11p ^= parental figure provided information when participants age 11.

### Use of psychiatric services

Table 4 shows the unadjusted associations between covariates and use of psychiatric services before age 11 and between age 11 and age 19. We found the expected associations between psychosocial covariates and use of psychiatric services. For example, compared to males, females show significantly lower odds (OR 0.38) of using services *before *age 11, but significantly higher odds (OR 2.07) of service use *after *age 11; including covariates made little difference to these results. Young people from reconstituted families and those with lack of parental care had a higher likelihood of using services, although his was not always statistically significant. With the exception of birth complications, most perinatal variables were again unrelated, or only marginally associated, with use of psychiatric services. Compared to those without birth complications, those with two or more birth complication have substantially increased odds (OR 2.3-3.8) of psychiatric contact, either before or after age 11, although in the adjusted model this was attenuated (OR 1.7-2.9), but still significant for service contact both before age 11 and for lifetime psychiatric service contact.

**Table 4:** Associations (odds ratios) between social, demographic, perinatal factors and use of psychological services.

	Psych service use before age-11		Psych service use after 11		ANY Psych service use	
			
Covariates	Unadjust	Adjusted	Unadjust	Adjusted	Unadjust	Adjusted
**Psychosocial**						

**Sex ^@11^**						
Male	1.00	1.00	1.00	1.00	1.00	1.00
Female	**0.38 (0.23-0.62)**	**0.35 (0.21-0.59)**	**2.07 (1.09-3.91)**	**2.11 (1.08-4.11)**	0.91 (0.53-1.56)	0.93 (0.56-1.56)
**Social class ^@11p^**						
Non-manual	1.00	1.00	1.00	1.00	1.00	1.00
Manual	1.22 **(**0.69-2.16**)**	1.34 (0.70-2.54)	1.44 **(**0.59-3.51**)**	1.23 **(**0.60-2.54**)**	1.43 (0.88-2.32)	1.15 (0.75-1.76)
**Family structure ^@15^**						
2-parent	1.00	1.00	1.00	1.00	1.00	1.00
1-parent,	1.05 **(**0.47-2.33**)**	0.96 (0.41-2.25)	**2.08 (0.99-4.39)**	**2.05 (0.99-4.25)**	1.47 (0.78-2.78)	1.48 (0.80-2.73)
reconstituted/Other	**2.00 (1.00-4.00)**	1.48 (0.68-3.23)	**2.24 (0.87-5.77)**	**2.93 (1.35-6.36)**	**2.15 **(**1.22-3.79**)	**2.61 **(**1.58-4.30**)
**Same sex part ^@19^**						
No	1.00	1.00	1.00	1.00	1.00	1.00
Yes	NIL	NIL	**7.10 (3.29-15.33)**	**7.34 (3.44-15.64)**	**3.49 **(**1.72-7.11**)	**3.42 **(**1.51-7.77**)
**Gender conform ^@15^**						
Gender typical	1.00	1.00	1.00	1.00	1.00	1.00
Gender conformist	0.50 **(**0.15-1.62**)**	0.42 (0.13-1.41)	0.85 **(**0.20-3.66**)**	0.86 **(**0.23-3.19**)**	0.80 (0.28-2.30)	0.92 (0.35-2.40)
Gender nonconformist	1.00 **(**0.43-2.32**)**	0.90 (0.38-2.10)	0.67 **(**0.27-1.68**)**	1.28 **(**0.51-3.19**)**	1.04 (0.55-1.96)	1.56 (0.83-2.94)
**Religion ^@11p^**						
Protestant	1.00	1.00	1.00	1.00	1.00	1.00
Roman Catholic	0.65 **(**0.38-1.13**)**	0.66 (0.37-1.17)	1.17 **(**0.54-2.54**)**	1.08 **(**0.54-2.16**)**	0.84 (0.45-1.60)	0.84 (0.48-1.47)
Other (Baptist, etc)	0.50 **(**0.18-1.42**)**	0.52 (0.18-1.51)	1.48 **(**0.42-5.28**)**	1.26 **(**0.43-3.70**)**	1.23 (0.60-2.51)	1.11 (0.55-2.24)
None/Atheist/Agnostic	1.40 **(**0.80-2.46**)**	1.16 (0.64-2.11)	1.19 **(**0.43-3.24**)**	1.38 **(**0.54-3.52**)**	0.83 (0.29-2.40)	1.05 (0.39-2.83)

**Area deprivation ^@11^**	0.97 **(**0.85-1.11**)**	0.91 (0.78-1.06)	0.91 **(**0.75-1.10**)**	0.97 **(**0.83-1.13**)**	0.89 (0.75-1.06)	0.95 (0.83-1.10)
**Lack of par Care ^@15^**	1.14 (0.98-1.32)	1.13 (0.97-1.32)	**1.26 (1.01-1.56)**	**1.29 (1.07-1.55)**	1.12 (0.881.42)	1.15 (0.92-1.45)
**Parental control ^@15^**	1.19 **(**0.95-1.50**)**	1.14 (0.90-1.44)	0.96 **(**0.68-1.35**)**	0.98 **(**0.73-1.32**)**	1.24 (0.94-1.63)	1.24 (0.97-1.57)

**Perinatal ^all @11p^**						

**Maternal parity**						
**0**	1.00	1.00	1.00	1.00	1.00	1.00
1 or more	**0.58 (0.37-0.91)**	0.93 (0.37-2.34)	0.91 **(**0.19-4.23**)**	0.80 **(**0.44-1.49**)**	1.21 (0.43-3.37)	0.69 (0.40-1.21)
**Family size **						
2	1.00	1.00	1.00	1.00	1.00	1.00
3 or more	**0.33 (0.20-0.54)**	**0.45 (0.20-1.02)**	0.46 **(**0.13-1.59**)**	0.66 **(**0.25-1.71**)**	0.38 (0.13-1.10)	**0.43 **(**0.21-0.88**)
**Birth weight (g)**						
<2500	1.43 **(**0.57-3.55**)**	1.40 (0.53-3.71)	0.74 **(**0.21-2.61**)**	0.90 **(**0.29-2.84**)**	1.14 (0.36-3.63)	1.36 (0.47-3.94)
2500-3249	1.38 **(**0.81-2.37**)**	1.42 (0.81-2.49)	0.66 **(**0.29-1.49**)**	0.67 **(**0.33-1.37**)**	0.92 (0.56-1.50)	0.98 (0.59-1.63)
3250-3749	1.00	1.00	1.00	1.00	1.00	1.00
3750+	1.49 **(**0.81-2.75**)**	1.43 (0.75-2.72)	0.70 **(**0.32-1.53**)**	0.58 **(**0.29-1.16**)**	0.93 (0.51-1.70)	0.88 (0.51-1.54)
**N birth complications**						
0	1.00	1.00	1.00	1.00	1.00	1.00
1	0.81 **(**0.41-1.61**)**	0.73 (0.36-1.50)	1.58 **(**0.75-3.31**)**	1.24 **(**0.66-2.34**)**	**1.92 **(**1.02-3.62**)	1.68 (0.92-3.10)
2 or more	**2.26 (1.34-3.82)**	**2.29 (1.30-4.03)**	2.63 (0.87-7.94)	1.67 **(**0.59-4.72**)**	**3.77 **(**1.83-7.76**)	**2.92 **(**1.45-5.88**)
**Birth spacing (prior)**						
Under 2 years	1.00	1.00	1.00	1.00	1.00	1.00
2-5 years	2.02 **(**0.84-4.84**)**	2.03 (0.83-4.99)	0.76 **(**0.28-2.11**)**	0.68 **(**0.28-1.64**)**	0.94 (0.48-1.82)	0.90 (0.46-1.76)
6+ years or singleton	2.14 (0.96-4.74)	1.76 (0.72-4.34)	**0.39 (0.17-0.88)**	0.56 **(**0.26-1.21**)**	0.61 (0.21-1.73)	0.91 (0.42-1.94)
**Birth spacing (later)**						
Under 2 years	1.00	1.00	1.00	1.00	1.00	1.00
2-5 years	0.71 **(**0.33-1.56**)**	0.71 (0.32-1.57)	0.37 **(**0.10-1.32**)**	0.38 **(**0.11-1.23**)**	0.57 (0.27-1.20)	0.56 (0.27-1.16)
6+ years or singleton	1.44 **(**0.79-2.62**)**	1.22 (0.49-3.04)	0.41 **(**0.08-1.99**)**	0.70 **(**0.33-1.49**)**	0.88 (0.32-2.42)	1.05 (0.62-1.76)
**Maternal age (years)**						
*15-19*	**2.22 (1.00-4.94)**	1.60 (0.63-4.03)	0.74 **(**0.14-4.07**)**	0.77 **(**0.12-4.78**)**	1.03 (0.37-2.91)	1.29 (0.57-2.93)
*20-24*	1.55 **(**0.87-2.75**)**	1.71 (0.92-3.18)	1.26 **(**0.52-3.03**)**	1.26 **(**0.55-2.85**)**	**1.74 **(**1.00-3.03**)	1.59 (0.93-2.72)
*25-29 *	1.00	1.00	1.00	1.00	1.00	1.00
*30-34*	1.64 **(**0.88-3.04**)**	1.48 (0.77-2.84)	1.06 **(**0.41-2.74**)**	0.95 **(**0.41-2.25**)**	1.61 (0.77-3.41)	1.53 (0.77-3.05)
*35-46*	0.52 **(**0.12-2.26**)**	0.48 (0.11-2.15)	0.74 **(**0.06-8.86**)**	0.77 **(**0.10-6.08**)**	0.91 (0.24-3.48)	0.97 (0.28-3.37)
**Breastfeeding**						
None	1.00	1.00	1.00	1.00	1.00	1.00
Less than 6 months	0.92 **(**0.52-1.60**)**	0.92 (0.50-1.69)	1.05 **(**0.46-2.37**)**	0.94 **(**0.50-1.77**)**	0.88 (0.42-1.84)	0.86 (0.43-1.72)
6 months or more	0.83 **(**0.43-1.59**)**	1.05 (0.50-2.21)	1.33 **(**0.61-2.89**)**	1.09 **(**0.56-2.15**)**	1.21 (0.59-2.48)	1.01 (0.55-1.86)

Significant odds ratios (p = 0.05 or lower) are shown in bold. Final sample with multiple imputation = 2157.

^@11 ^= participant provided information at age 11; ^@15 ^= information provided at age 15; ^@19 ^= information provided at age 19; ^@11p ^= parental figure provided information when participants age 11.

## Discussion

This study demonstrated a significant and sizable association between the number of birth complications and psychiatric service contact. If replicated this is important, because within the current perinatal circumstances literature birth complications is a relatively new focus of study of substantial effect size, compared to other perinatal factors. As expected we demonstrated a significant association between adverse mental health outcomes and adolescent psychosocial variables such as reconstituted family structure, perceived lack of parental care and having a same sex partner. Previous observations regarding perinatal variables were not replicated, but given the currently limited evidence-base, consisting of a handful of large population based studies, there are a number of potential explanations for the general lack of association with mental health outcomes.

### Associations between birth complications and psychiatric service use

A significant association was observed between birth complications and contact with psychiatric services before the age of 11. This is consistent with previous work linking birth trauma and developmental disorders [32], although the potential influence of parental behaviour should be borne in mind and this is discussed in detail later. Although still somewhat controversial, several authors link the concepts of *general *neurological, potentially birth-related damage, or perinatal stress to the development of psychiatric or mental health problems using a variety of terms such as *Minimal Brain Dysfunction *(see review by Rutter [33]) and more recently, *deficits in attention, motor control, perception *(DAMP). In relation to DAMP the attention has focused on learning and language difficulties, autistic spectrum and attention deficit/hyperactivity (ADHD) disorders, although more general psychopathology such as anxiety and depression have also been linked [34].

No relationship was observed between birth complications and suicidality in mid teens, although a statistically non-significant association was observed between such complications and NSSI at the age of 19, an association which increased following adjustment for background factors, suggesting that this perinatal variable may be a risk mediator. Such (weak) associations are consistent with well established links between obstetric complications and increased risk of adult psychosis [35], although links with affective disorders [36] or suicidality [37] have not been clearly established. We find a general association between obstetric complications and a crude measure of psychiatric problems (service contact), but any association may be stronger among specific types of psychiatric disorder; a future study will look at the associations between birth complication more specific psychiatric disorders and symptoms, such as ADHD.

### Lack of associations with perinatal circumstances and mental heath outcomes

Objectively, we found few associations between perinatal circumstances and mental health outcomes and can only speculate as to the reasons for the predominantly null findings. One explanation for the lack of statistically significant associations with perinatal variables may be the smaller power in this study compared with population based studies. This may be especially relevant for groups such as those with birth weights below 2500g, where the case numbers were small. The data do not indicate any consistent trend towards the risks associated with perinatal variables being attenuated on multivariable analysis, suggesting that controlling for adolescent psychosocial factors does not account for this lack of association.

An alternative explanation may be provided by the equalisation in youth hypothesis which suggests that during adolescence young people with different socioeconomic backgrounds demonstrate relative parity of health [38], it being only later in life that health inequalities (re)emerge. Whereas other studies on perinatal influences followed up subjects to their fourth decade, this study has, (so far) not collected any outcome data beyond early adulthood.

A notable exception to the apparent lack of perinatal influence is the case of young maternal age, for which the data does suggest a trend towards an attenuation of the associated risks following adjustment. This suggests that previously reported associations may have been mediated, in part at least, by more proximal adolescent psychosocial factors such as family structure and parental relationships.

### Associations between psychosocial circumstances and mental health outcomes

Those from a single parent family were at greater risk than those living with two biological parents, although such differences were not statistically significant and there was a trend for these risks to attenuate after adjustment for other factors. Those from a "reconstituted family" were at significantly greater risk, although this also attenuated somewhat following adjustment. These observations appear consistent with a recent New Zealand study, which concluded that the associations between exposure to a single parent family and adversity can be attributed to the social environment [20]. In contrast to family structure, perceived deficiency in parental care was associated with increased risk which was minimally attenuated following adjustment. These observations seem consistent with other studies which suggest it is the quality and stability of the parent child relationship or family function which is the risk mediator [19,20,39], whereas family structure is a more distal risk factor.

The relationships between same sex partner and suicidality, although statistically significant only in the case of NSSI at age 19, are attenuated following adjustment. This is consistent with the more likely interpretation of sexual orientation as a risk indicator rather than mediator i.e. the increased psychiatric risk is mediated by associated sociological, psychological and cultural factors, such as victimisation or identity issues.

The association between mental health outcome and same sex partner is consistent with previous studies on the relationship between mental health and homosexuality. While such an association between sexual orientation and risk of suicide or NSSI is well-established [40,41], another robust association is that between birth order (specifically the number of older brothers) and male homosexual orientation [42]. The leading explanation for this association is the H-y antigen theory [43], which posits that a progressively stronger immunological response to each male foetus within the intrauterine environment plays a role in determining sexual orientation. Previously reported associations between birth order and suicidality [13,14] may therefore be mediated by sexual orientation, with birth order being merely a risk indicator. However, in the absence of any apparent relationship in this study, between maternal parity and outcome, even before adjustment, it is not possible to comment on the extent to which sexual orientation influences our findings, although the 'elder brother effect' may have confounded previous observations on maternal parity.

### Limitations of study

Much of the data is self reported, and so it may be subject to recall bias. The data available on parental care and control, and on family structure, was cross sectional information, which was collected at age 15. Length of exposure to such factors during the course of childhood was not available, and so not available for analysis. Although the study provided a rich dataset, the power of the study to identify associations between perinatal circumstances and mental health outcomes was limited, and this should be borne in mind in the interpretation of the lack of association in the present work.

Our study focused on a limited set of narrowly operationalized outcomes, potentially losing insight about the link between perinatal circumstances and severity of psychiatric symptoms and specific psychiatric disorders; further work to explore these links is required. Data involving the use of psychiatric services, especially before the age of 11, ought to be interpreted with some caution as these variables may be greatly influenced by parental attitude and behaviour and lack sensitivity when measuring the degree of psychiatric symptomatology. It therefore may not be a valid measure of prevalence of mental disorders. For example, parents with small families may be more likely to seek psychiatric help for their young children than are those of large families. Observations of relationships between service use and variables such as family size, pregnancy spacing and maternal parity, should be interpreted with this in mind. However, data on suicidal ideation, suicide attempt and NSSI, although self reported, are not likely to be influenced by parental behaviour as it is would only be indirectly influenced by parental help-seeking.

The data available on parental care and control, and on family structure, was cross sectional information, which was collected at age 15. Length of exposure to such factors during the course of childhood was not available, and so not available for analysis. Although the study provided a rich dataset, the power of the study to identify associations between perinatal circumstances and mental health outcomes was limited, and this should be borne in mind in the interpretation of the lack of association in the present work.

## Conclusion

A number of adolescent psychosocial variables may be risk factors for suicidality which could be the focus of preventative measures, the most notable being the quality of parent child relationships. Further research is necessary to clarify the role of variables such as sexual orientation and birth trauma in the development of suicidal behaviour. Although we echo Tharpar & Rutter's [21] caution in ascribing perinatal factors a causal role in the development of psychiatric problems, the strong relationship we find between obstetric complications in this population sample and psychiatric service use, which adjusts for a range of likely confounds, re-opens the debate in this area.

## Competing interests

RY is supported financially by the Medical Research Council of Great Britain at the Social and Public Health Sciences Unit as part of the Youth and Health Programme (WBS U.1300.00.007). The authors declare no other competing interests.

## Authors' contributions

All authors contributed to the writing of the manuscript and jointly conceived the theoretical approach taken in the paper. RY participated in design and data collection of the final phase of the 11-16/16+ study, and performed the statistical analysis. Conceptually VR and CS primarily focused on perinatal and RY the social influences. All authors read and approved the final manuscript.

## Pre-publication history

The pre-publication history for this paper can be accessed here:

http://www.biomedcentral.com/1471-2458/11/875/prepub

## References

[B1] Rutter M Environmentally mediated risks for psychopathology: research strategies and findings J Am Acad Child Psychiatry 2005 44 1 3 18 10.1097/01.chi.0000145374.45992.c9 15608539

[B2] Rutter M How the environment affects mental health Br J Psychiatry 2005 186 1 4 6 10.1192/bjp.186.1.4 15630116

[B3] Bowlby J The making and breaking of affectional bonds. I. Aetiology and psychopathology in the light of attachment theory. An expanded version of the Fiftieth Maudsley Lecture, delivered before the Royal College of Psychiatrists, 19 November 1976 Br J Psychiatry 1977 130 201 210 10.1192/bjp.130.3.201 843768

[B4] Richards M Hardy R Wadsworth M The effects of divorce and separation on mental health in a national UK birth cohort Psychol Med 1997 27 5 1121 1128 10.1017/S003329179700559X 9300516

[B5] Browne A Finkelhor D Impact of Child Sexual Abuse - a Review of the Research Psychol Bull 1986 99 1 66 77 3704036

[B6] Scott KM Smith DR Ellis PM Prospectively Ascertained Child Maltreatment and Its Association With DSM-IV Mental Disorders in Young Adults Arch Gen Psychiatry 2010 67 7 712 719 10.1001/archgenpsychiatry.2010.71 20603452

[B7] Mullen PE Martin JL Anderson JC Romans SE Herbison GP Childhood sexual abuse and mental health in adult life Br J Psychiatry 1993 163 721 732 10.1192/bjp.163.6.721 8306113

[B8] Enns MW Cox BJ Afifi TO De Graaf R Ten Have M Sareen J Childhood adversities and risk for suicidal ideation and attempts: a longitudinal population-based study Psychol Med 2006 36 12 1769 1778 10.1017/S0033291706008646 16999880

[B9] Lipschitz DS Winegar RK Nicolaou AL Hartnick E Wolfson M Southwick SM Perceived abuse and neglect as risk factors for suicidal behavior in adolescent inpatients J Nerv Ment Dis 1999 187 1 32 39 10.1097/00005053-199901000-00006 9952251

[B10] Brown AS van Os J Driessens C Hoek HW Susser ES Further Evidence of Relation Between Prenatal Famine and Major Affective Disorder Am J Psychiatry 2000 157 2 190 195 10.1176/appi.ajp.157.2.190 10671386

[B11] O'Keane V Scott J From 'obstetric complications' to a maternal-foetal origin hypothesis of mood disorder Br J Psychiatry 2005 186 367 368 10.1192/bjp.186.5.367 15863739

[B12] Thompson C Syddall H Rodin I Osmond C Barker DJ Birth weight and the risk of depressive disorder in late life Br J Psychiatry 2001 179 450 455 10.1192/bjp.179.5.450 11689404

[B13] Gravseth HM Mehlum L Bjerkedal T Kristensen P Suicide in young Norwegians in a life course perspective: population-based cohort study J Epidemiol Community Health 2010 64 5 407 412 10.1136/jech.2008.083485 19679707

[B14] Riordan DV Selvaraj S Stark C Gilbert JS Perinatal circumstances and risk of offspring suicide. Birth cohort study Br J Psychiatry 2006 189 502 507 10.1192/bjp.bp.105.015974 17139033

[B15] Mittendorfer-Rutz E Rasmussen F Wasserman D Restricted fetal growth and adverse maternal psychosocial and socioeconomic conditions as risk factors for suicidal behaviour of offspring: a cohort study Lancet 2004 364 9440 1135 1140 10.1016/S0140-6736(04)17099-2 15451220

[B16] Barker DJ Osmond C Rodin I Fall CH Winter PD Low weight gain in infancy and suicide in adult life BMJ 1995 311 7014 1203 10.1136/bmj.311.7014.1203 7488897 PMC2551118

[B17] Tsankova N Renthal W Kumar A Nestler EJ Epigenetic regulation in psychiatric disorders Nat Rev Neurosci 2007 8 5 355 367 17453016 10.1038/nrn2132

[B18] Chase-Lansdale PL Cherlin AJ Kiernan KE The long-term effects of parental divorce on the mental health of young adults: a developmental perspective Child Dev 1995 66 6 1614 1634 10.2307/1131900 8556889

[B19] O'Connor TG Dunn J Jenkins JM Pickering K Rasbash J Family settings and children's adjustment: differential adjustment within and across families Br J Psychiatry 2001 179 110 115 10.1192/bjp.179.2.110 11483471

[B20] Fergusson DM Boden JM Horwood LJ Exposure to single parenthood in childhood and later mental health, educational, economic, and criminal behavior outcomes Arch Gen Psychiatry 2007 64 9 1089 1095 10.1001/archpsyc.64.9.1089 17768274

[B21] Thapar A Rutter M Do prenatal risk factors cause psychiatric disorder? Be wary of causal claims Br J Psychiatry 2009 195 2 100 101 10.1192/bjp.bp.109.062828 19648537

[B22] West P Sweeting H Background, Rationale and Design of the West of Scotland 11 to 16 study Working Paper No 53 1996 Glasgow: MRC Social & Public Health Sciences Unit

[B23] Ecob R West P Sweeting H The West of Scotland 11 to 16 Study: schools, sample design and implementation issues Working Paper No 61 1996 Glasgow: MRC Medical Sociology Unit

[B24] West P Sweeting H Der G Barton J Lucas C Voice-DISC identified DSM-IV disorders among 15-year-olds in the west of Scotland J Am Acad Child Adolesc Psychiatry 2003 42 8 941 949 10.1097/01.CHI.0000046907.27264.E4 12874496

[B25] Sweeting H Der G West P Bias, attrition and weighting in respect of the West of Scotland 11 to 16 Study's baseline, S2 and S4 surveys Working Paper No 9 2001 Glasgow: MRC Social & Public Health Sciences Unit

[B26] Section on Breastfeeding Breastfeeding and the Use of Human Milk Pediatrics 2005 115 2 496 506 15687461 10.1542/peds.2004-2491

[B27] McLoone P Carstairs scores for Scottish postcode sectors from the 2001 Census 2004 Glasgow: MRC Social and Public Health Sciences Unit

[B28] ONS Standard Occupational Classification 2000 2 London: The Stationery Office

[B29] Klimidis S Minas IH Ata AW The PBI-BC: A brief current form of the Parental Bonding Instrument for adolescent research Compr Psychiatry 1992 33 6 374 377 10.1016/0010-440X(92)90058-X 1451449

[B30] Young R Sweeting H Adolescent bullying, relationships, psychological well-being, and gender-atypical behavior: A gender diagnosticity approach Sex Roles 2004 50 7-8 525 537

[B31] Lippa RA Gender-related traits in gay men, lesbian women, and heterosexual men and women: the virtual identify of homosexual-heterosexual diagnosticity and gender diagnosticity J Pers 2000 68 5 899 926 10.1111/1467-6494.00120 11001153

[B32] Eaton WW Mortensen PB Thomsen PH Frydenberg M Obstetric complications and risk for severe psychopathology in childhood J Autism Dev Disord 2001 31 3 279 285 10.1023/A:1010743203048 11518482

[B33] Rutter M Syndromes Attributed to Minimal Brain-Dysfunction in Childhood Am J Psychiat 1982 139 1 21 33 6119908 10.1176/ajp.139.1.21

[B34] Gillberg C Deficits in attention, motor control, and perception: a brief review Arch Dis Child 2003 88 10 904 910 10.1136/adc.88.10.904 14500312 PMC1719331

[B35] Cannon M Jones PB Murray RM Obstetric complications and schizophrenia: historical and meta-analytic review Am J Psychiatry 2002 159 7 1080 1092 10.1176/appi.ajp.159.7.1080 12091183

[B36] Scott J McNeill Y Cavanagh J Cannon M Murray R Exposure to obstetric complications and subsequent development of bipolar disorder: Systematic review Br J Psychiatry 2006 189 3 11 10.1192/bjp.bp.105.010579 16816299

[B37] Neugebauer R Reuss ML Association of maternal, antenatal and perinatal complications with suicide in adolescence and young adulthood Acta Psychiatr Scand 1998 97 6 412 418 10.1111/j.1600-0447.1998.tb10024.x 9669512

[B38] West P Health inequalities in the early years: Is there equalisation in youth? Soc Sci Med 1997 44 6 833 858 10.1016/S0277-9536(96)00188-8 9080566

[B39] Weich S Patterson J Shaw R Stewart-Brown S Family relationships in childhood and common psychiatric disorders in later life: systematic review of prospective studies Br J Psychiatry 2009 194 5 392 398 10.1192/bjp.bp.107.042515 19407266

[B40] Skegg K Nada-Raja S Dickson N Paul C Williams S Sexual orientation and self-harm in men and women Am J Psychiatry 2003 160 3 541 546 10.1176/appi.ajp.160.3.541 12611836

[B41] Fergusson DM Horwood LJ Beautrais AL Is sexual orientation related to mental health problems and suicidality in young people? Arch Gen Psychiatry 1999 56 10 876 880 10.1001/archpsyc.56.10.876 10530626

[B42] Blanchard R Bogaert AF Homosexuality in men and number of older brothers Am J Psychiatry 1996 153 1 27 31 8540587 10.1176/ajp.153.1.27

[B43] Blanchard R Fraternal birth order and the maternal immune hypothesis of male homosexuality Horm Behav 2001 40 2 105 114 10.1006/hbeh.2001.1681 11534970

